# Serological evidence of virus infection in *Eidolon helvum* fruit bats: implications for bushmeat consumption in Nigeria

**DOI:** 10.3389/fpubh.2023.1283113

**Published:** 2023-11-27

**Authors:** Diego Cantoni, Martin Mayora-Neto, Mariliza Derveni, Kelly da Costa, Joanne Del Rosario, Veronica O. Ameh, Claude T. Sabeta, Bethany Auld, Arran Hamlet, Ian M. Jones, Edward Wright, Simon D. Scott, Efstathios S. Giotis, Ashley C. Banyard, Nigel Temperton

**Affiliations:** ^1^Viral Pseudotype Unit, Medway School of Pharmacy, Universities of Kent and Greenwich, Chatham, United Kingdom; ^2^Viral Pseudotype Unit, School of Life Sciences, University of Sussex, Brighton, United Kingdom; ^3^Department of Veterinary Public Health and Preventive Medicine, College of Veterinary Medicine, Federal University of Agriculture Makurdi, Makurdi, Nigeria; ^4^Department of Veterinary Tropical Diseases, Faculty of Veterinary Science, University of Pretoria, Onderstepoort, South Africa; ^5^World Organisation for Animal Health Rabies Reference Laboratory, Agricultural Research Council-Onderstepoort Veterinary Research, Onderstepoort, South Africa; ^6^Department of Infectious Disease Epidemiology, MRC Centre for Global Infectious Disease Analysis, Imperial College London, London, United Kingdom; ^7^School of Biological Sciences, University of Reading, Reading, United Kingdom; ^8^Department of Infectious Diseases, Imperial College London, London, United Kingdom; ^9^School of Life Sciences, University of Essex, Colchester, United Kingdom; ^10^Animal and Plant Health Agency, Weybridge, United Kingdom

**Keywords:** *Eidolon helvum*, pseudotypes, Ebola virus, Nipah virus, Marburg virus, henipavirus, H17N10, Ghana bat henipavirus

## Abstract

**Introduction:**

The *Eidolon helvum* fruit bat is one of the most widely distributed fruit bats in Africa and known to be a reservoir for several pathogenic viruses that can cause disease in animals and humans. To assess the risk of zoonotic spillover, we conducted a serological survey of 304 serum samples from *E. helvum* bats that were captured for human consumption in Makurdi, Nigeria.

**Methods:**

Using pseudotyped viruses, we screened 304 serum samples for neutralizing antibodies against viruses from the *Coronaviridae, Filoviridae, Orthomyxoviridae* and *Paramyxoviridae* families.

**Results:**

We report the presence of neutralizing antibodies against henipavirus lineage GH-M74a virus (odds ratio 6.23; *p* < 0.001), Nipah virus (odds ratio 4.04; *p* = 0.00031), bat influenza H17N10 virus (odds ratio 7.25; *p* < 0.001) and no significant association with Ebola virus (odds ratio 0.56; *p* = 0.375) in this bat cohort.

**Conclusion:**

The data suggest a potential risk of zoonotic spillover including the possible circulation of highly pathogenic viruses in *E. helvum* populations. These findings highlight the importance of maintaining sero-surveillance of *E. helvum,* and the necessity for further, more comprehensive investigations to monitor changes in virus prevalence, distribution over time, and across different geographic locations.

## Introduction

Throughout the course of human history, viral zoonotic spillover events have been sporadic and sometimes catastrophic, resulting in several highly lethal pandemics (1). With approximately 60–75% of all human infectious diseases arising from zoonotic transmission (2), it is crucial to remain vigilant in identifying and monitoring potential sources of zoonotic spillover, such as wildlife reservoirs, in order to prevent future outbreaks. Over the past four decades, bats have been identified as a significant source of zoonotic events that have sparked major outbreaks of viruses with considerable implications for human health (3). As the second most diverse mammalian order, bats have been linked to the transmission of a range of viruses, including coronaviruses, filoviruses, lyssaviruses, and henipaviruses, among others (4, 5). With over 12,000 bat-derived virus sequences spanning 30 viral families, having relevance to both veterinary and medical sectors, the need for surveillance of bat populations is critical to assess and mitigate the risk of potential zoonotic spillover events.

*Eidolon helvum*, the straw-colored fruit bat, is one of the most widely distributed fruit bats in Africa (6). It has extensive migratory patterns of over 2,000 kilometers and is hypothesized to migrate based on availability of fruits to increase reproductive success (7, 8). According to the DBatVir database (9) (accessed on 25th August, 2023) 17 different viruses, including *Coronaviridae* and *Paramyxoviridae*, have been detected in *E. helvum*.

In several countries within Africa, bats, including *E. helvum*, are often hunted for either bushmeat (10, 11) or as a form of pest control to mitigate fruit farming losses (12). One of these countries is Nigeria, where *E. helvum* can be found in bushmeat markets to be sold as food (12–15) or as resources for traditional medicine (16). Though many of these practices have been limited by policies imposed due to the Ebola outbreak in 2014 (17), continued bat hunting for human consumption greatly increases the risk of zoonotic transmission, making surveillance vital for public health and wildlife conservation efforts in the region.

Although serological evidence of neutralizing antibodies in bat sera is not definitive proof of active virus infection in bats, it does suggest that bats have been exposed to the virus or closely related viruses, and have mounted an adaptive immune response. Screening for neutralizing antibodies in bats using highly pathogenic viruses requires use of high containment facilities, which can be avoided by using pseudotyped viruses (PVs) in neutralization assays as they are considered safe for handling under biosafety level 2 conditions. Pseudotyped virus neutralization assays (PVNA) have gained widespread usage for detecting neutralizing antibodies due to their high sensitivity and robust correlation with live virus neutralization assays (18).

This study aimed to preliminarily assess the potential presence of pathogenic viruses in bats hunted for bushmeat, to allow further, more comprehensive follow-up investigations focusing on the presence of animal and human pathogenic viruses in Nigeria. We screened 304 serum samples from *E. helvum* bats that were captured for human consumption in Makurdi, Nigeria using PVs expressing the viral glycoproteins of several viruses known to pose high public health risks (Table 1). Due to limited volumes of sera, we prioritized the order of screening based on the viruses’ potential risk to animal and human health (19–21). Our findings indicate the presence of neutralizing antibodies against important representatives of different virus families, which may suggest the circulation of several highly pathogenic viruses with pandemic potential, or closely related viruses, in colonies of *E. helvum* in Nigeria, and therefore, warrants further comprehensive research.

**Table 1:** List of viruses pseudotyped for screening *E. helvum* samples in this study.

Virus family	Genus	Species	
*Coronaviridae*	*Betacoronavirus*	Severe acute respiratory virus 1	(SARS-CoV-1)
		Severe acute respiratory virus 2	(SARS-CoV-2)
		Bat coronavirus RaTG13	(RaTG13)
*Filoviridae*	*Ebolavirus*	Zaire ebolavirus	(EBOV)
	*Marburgvirus*	Marburg virus	(MARV)
*Orthomyxoviridae*	*Alphainfluenzavirus*	Bat influenza H17N10	(H17N10)
*Paramyxoviridae*	*Henipavirus*	Nipah virus	(NiV)
		Ghanaian henipavirus M74a	(GH-M74a)

## Materials and methods

### Bat sera collection

All sera were collected from terminally bled straw-colored fruit bats (*Eidolon helvum*) that were captured for human consumption in Makurdi, Benue State Nigeria (7^o^44′25.7″N 8^o^31′52.8″E). The bats in Makurdi were collected from roosts in trees in and around the Benue State Government House, and on trees in private residences close to the government house. Bat roost sites and seasons when bats roost in Makurdi were identified by collaborating and interacting with local bat hunters. Convenient sampling was done by collecting samples only on days when bat hunters set traps to capture bats. Bat roost sites were visited once every week over a period of 12–14 weeks. Personnel protective equipment with respirators were worn by those involved in the sampling of bats captured for human consumption. The bats were captured by setting mist nets in the evenings when they went out to feed. The bats were trapped in the nets as they return to their roost sites on trees during the early hours of the following morning. The bats were then carefully taken down alive from the nets by the hunters, identified morphologically using phenotypic properties and bled from the brachial vein, using a 5 mL syringe and a 21-gauge hypodermic needle by the investigator. Bats were also sampled from roosts on trees in private residences where permissions were gained. Sampling was carried out for two consecutive seasons (November 2017–March 2018 and November 2018–March 2019). The whole blood samples were placed in well-labeled 7 mL tubes. These were placed on ice in cooler boxes and transported to the Amadu Ali Centre for Public Health and Comparative Medicine Laboratory, Federal University of Agriculture Makurdi. Sera were harvested from whole blood by centrifuging at 3,000 revolutions per minute for 10 min. Serum samples were placed in labeled 2 mL screwcap cryovials, heat-inactivated at 56°C for 30 min and stored at −20°C, then later transported to the Animal and Plant Health Agency, Weybridge (United Kingdom) after an import license was approved (License Number: ITIMP19.1408). Ethical approval for this research study was granted by the Animal Ethics Committee and Research Ethics Committee of University of Pretoria (certificate numbers V092-18 and REC097-18). The Director/Chief Veterinary Officer of Nigeria, Department of Veterinary and Pest Control Services, Federal Ministry of Agriculture and Pest Control Services, Abuja Nigeria granted permission to sampling of bat populations (license number VDS/194/S.4/11/T/85).

### Pseudotype virus production

Lentiviral (HIV) based pseudotypes used for this study were generated and characterized as described in detail, Influenza H17 (22), SARS-CoV-1 (23), SARS-CoV-2 (24), RaTG13 (25), EBOV and MARV (26), while NiV and GH-M74a were adapted from Khetawat et al. (27). Briefly, 1.0 μg of p8.91 plasmid encoding the HIV gag-pol was mixed with 1.5 μg of pCSFLW reporter gene and 1 μg of each surface viral glycoproteins as described in the studies cited in the previous sentence and in Table 2. After mixing the plasmids in 200 μL of Opti-MEM (ThermoFisher, Woolwich, UK), Fugene HD (Promega, Southampton, UK) transfection reagent was added at a 1:3 (plasmid:Fugene HD) ratio and incubated for 15 min prior to adding the transfection complexes to HEK293T/17 cells in T-75 cell culture flasks. Pseudotypes were harvested at 48- and 72-h post transfection, whereby culture media was removed from the flasks and filtered through a 0.45 μM cellulose acetate filter (Corning, Deeside, UK). Samples were aliquoted and frozen at −80°C for long term storage prior to use.

**Table 2:** Plasmids used to generate pseudotyped viruses.

Viral glycoprotein	Accession	Vector	Target cells
**Coronaviridae**			
SARS-CoV S (Tor2)	NC_004718.3	pcDNA 3.1+	HEK293T ACE2 + TMPRSS2
SARS-CoV-2 S (Wuhan)	NC_045512.2	pcDNA 3.1+	HEK293T ACE2 + TMPRSS2
RaTG13 S	QHR63300.2	pcDNA 3.1+	HEK293T ACE2 + TMPRSS2
**Filoviridae**			
EBOV Mayinga 76 GP	EU224440	pCAGGS	CHO
MARV Angola 05 GP	DQ447660	pCAGGS	CHO
**Orthomyxoviridae**			
IAV-like H17N10 H17	AFC35438.1	pI.18	MDCK II
**Paramyxoviridae**			
NiVb G and F	JN808864.1	pCAGGS	BHK
GH-M74A G and F	HQ660129	pCAGGS	BHK

HEK293T cells were transfected with Angiotensin-converting enzyme 2 (ACE2) and Transmembrane protease, serine 2 (TMPRSS2). CHO, Chinese hamster ovary cells; MDCK, Madin-Darby canine kidney cells. For this study we used the G and F genes from a Bangladeshi NiV isolate (NiVb).

### Pseudotype virus titration

Pseudotyped viruses were titrated by serially diluting filtered pseudotypes with fresh DMEM starting from a 1:2 to a final 1:512 dilution using white 96-well flat bottom plates (ThermoFisher, Woolwich, UK). Then appropriate target cells that allow permissibility for infection were added (Table 2) and plates were returned to the incubator. After 48 h, cells were lysed with Bright-Glo reagent (Promega, Southampton, UK), and luciferase expression levels were assessed using a GloMax plate reader (Promega, Southampton, UK).

### Sera screen and neutralizations

Sera were initially screened at single point dilution (1:100, except NiV and GH-M74a at 1:50). Each plate had wells containing PV only to determine maximum pseudotype entry. Samples where a 1 log decrease in RLU compared to the no-serum virus-only control was observed were then selected for cytotoxicity assay using Cell Titre Glo kit (Promega, Southampton, UK) and light microscopy to verify viability of cells prior to undertaking PVNA (data not shown). Neutralization assays were carried out by mixing bat sera, positive control sera (EBOV: WHO International Standard for Ebola virus Antibodies, 15/262, H17: mouse polyclonal antibodies raised to purified H17 antigen expressed using the baculovirus system as described in Loureiro et al. (28) and Shelton et al. (29), or positive monoclonal antibody NiV: m102.4 (30), with cell culture media at a starting input of 1:40 ratio (sera: cell culture media) and diluted either 4-fold (GH-M74a and NiV) to 1:5,000 or 8-fold (all other viruses) to 1:5120. PVs were added to the plates at a minimum RLU input of 10^5^ per well, incubated for 1 h at 37°C, followed by addition of target cells at a density of 10^4^ cells per well, except for NiV and GH-M74a where a cell density of 20^4^ cells per well was used. Plates were incubated at 37°C and 5% CO_2_ for 48 h prior to lysis using Bright-Glo reagent according to manufacturer’s protocol and monitoring of luciferase expression using a GloMax plate reader. Regression curves were fitted using GraphPad Prism 8 software (San Diego, CA, United States) as described previously (31). Some PVNAs tested fewer bat serum samples, due to the limited volumes supplied for this study. Currently, no positive control reagent exists for GH-M74a.

### Statistical analysis

Data were analyzed using STATA version 16 (StataCorp, College Station, TX, United States). Descriptive statistics were used to summarize the distribution of the variables. Bivariate logistic regression was used to determine the association between the presence or absence of neutralizing antibodies against each virus. Multivariable logistic regression was used to adjust for confounding factors. The data were coded as binary variables (1 = positive, 0 = negative). Logistic regression was used to determine the association between the presence or absence of neutralizing antibodies against each virus and the dependent variable was the presence or absence of neutralizing antibodies against each virus. Crude and adjusted odds ratios (OR) were estimated with 95% confidence intervals (CI) to measure the strength of association between each variable and the presence or absence of neutralizing antibodies.

## Results

**Figure 1:**
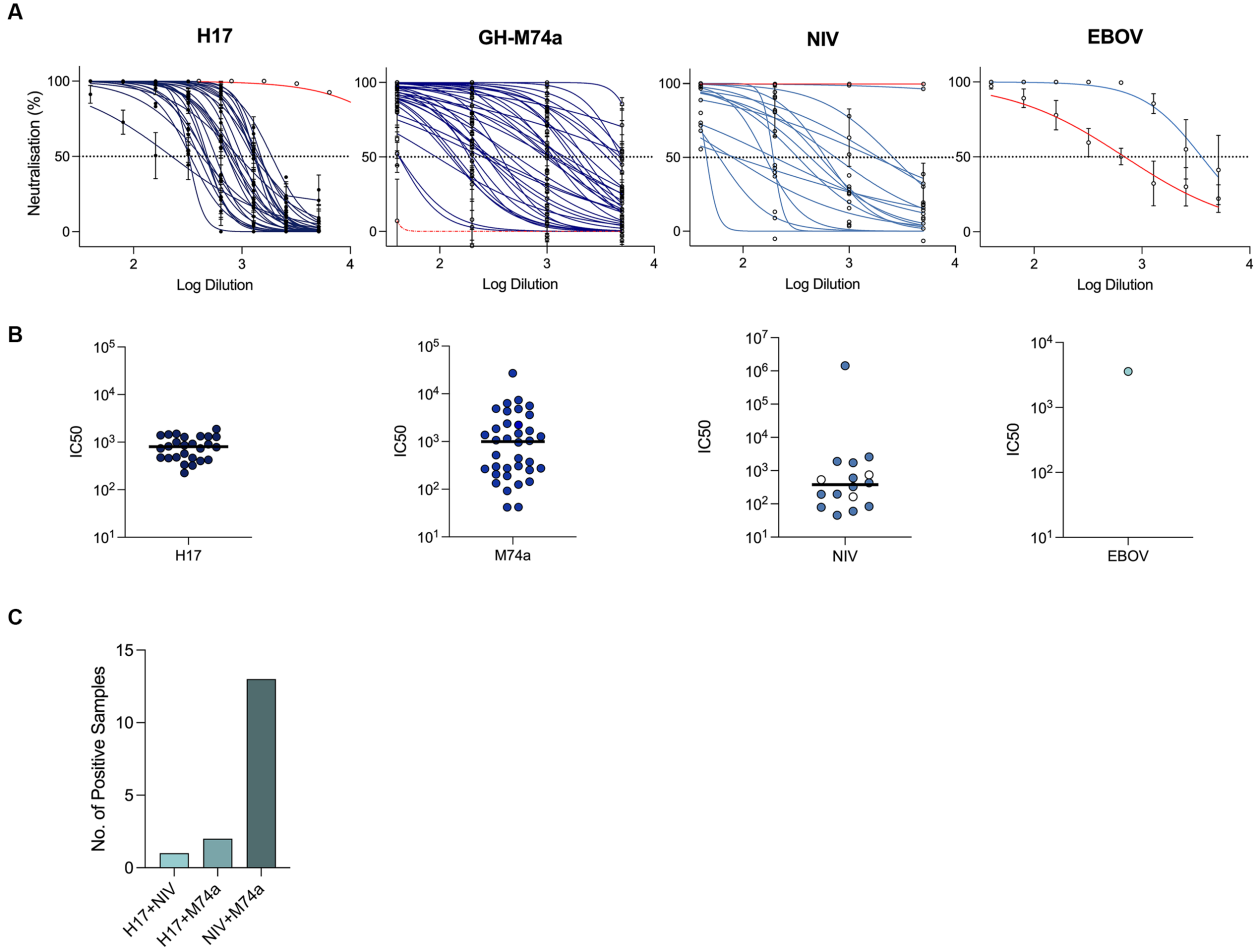
Positive detection of neutralizing antibodies in *E. helvum* by PVNA assay. Neutralization curves derived from pMN assays against positive samples that were selected from the initial screening, red curves denoting positive control reagents, with the exception of GH-M-74a which was not neutralized by NiV 102.4 mAb (red dotted line) **(A)**. IC50s calculated by pMN assays against each PV **(B)**. In the panel displaying samples positive for NiV, empty white circles denote samples that did not cross-neutralize GH-M74a. Number of samples positive for neutralizing antibodies against multiple viruses **(C)**.

Our PVNA screening revealed the presence of neutralizing antibodies against several of the pseudotyped viruses tested (Figures 1A,B; Table 3). However, no neutralization was observed against the *Coronaviridae* members SARS-CoV-2, SARS-CoV, and the bat coronavirus RaTG13. Although neutralizing antibodies against EBOV PVs were detected in a single sample (*n* = 1/278), no neutralization was observed against MARV. We also found several samples positive for neutralizing antibodies against influenza A virus H17N10 PVs (*n* = 26/304), indicating the presence of positive or cross-reactive H17 neutralizing antibodies. Additionally, positive samples were detected for Nipah virus (NiV) (*n* = 16/54) and GH-M74a (*n* = 36/54) from the *Paramyxoviridae* PVs.

**Table 3:** Results of pMN assays with each PV.

Viruses	Positive samples	Negative	Percentage
**Coronaviridae**			
SARS-CoV-1	0	304	0.00%
SARS-CoV-2	0	290	0.00%
RaTG13	0	290	0.00%
**Filoviridae**			
EBOV	1	278	0.36%
MARV	0	279	0.00%
**Orthomyxoviridae**			
H17N10	26	304	8.55%
**Paramyxoviridae**			
NiV	16	54	29.63%
Gh-M74a	36	54	66.67%

Sample sizes decreased due to limited sera volumes.

Our logistic regression analysis shows that the presence of neutralizing antibodies is significantly associated with virus type, as indicated by the *p*-values for the virus coefficients. Specifically, the odds of samples having neutralizing antibodies are significantly higher for H17 (odds ratio = 7.25, *p* < 0.001), NiV (odds ratio = 4.04, *p* = 0.00031), and GH-M74a (odds ratio = 6.23, *p* < 0.001) compared to the reference category of SARS-CoV-2. EBOV did not show a significant association with neutralizing antibody status (odds ratio = 0.56, *p* = 0.375).

We further analyzed the data to investigate the presence of samples positive for neutralizing antibodies against multiple viruses (Figure 1C). Our analysis revealed one sample positive for H17 and NiV, two samples positive for H17 and GH-M74a, and 13 samples positive for both NiV and GH-M74a. These findings suggest that multiple viruses may have circulated within the same bat or that antibodies to one of the three viruses may cross-neutralize against multiple viruses.

## Discussion

This study investigated the seroprevalence of neutralizing antibodies against several highly pathogenic viruses in *E. helvum* bats in Nigeria. We report a high level of seroprevalence against GH-M74a (66.7% of samples) and NiV (29.6%). The high level of seropositivity against GH-M74a could be the result of a recent resolved infection or the presence of multiple pathogens circulating with similar antigenicity. Given that the two viruses share a key conserved region in their attachment protein, and that GH-M74a antibodies have been shown to cross neutralize NiV (32), the significance of our study becomes evident when we consider the detection of three unique samples that, notably, exhibited neutralization exclusively against NiV but not GH-M74a. This suggests the potential existence of a closely related virus to NiV, or, though unlikely, the presence of NiV, which has yet to be identified in bats in Africa. In any case, the predictive value of a virus’s capability to utilize highly conserved receptors is significant when assessing the potential for viral emergence and cross-species transmission (33) Therefore, our results may indicate the presence of a closely related virus whose pathogenicity and virulence remain to be characterized. The henipavirus designated Ghana virus (GhV) remains the only African henipavirus that has been fully sequenced, isolated from *E. helvum* (34). However, Henipavirus and henipa-like virus antibodies have been detected in *E. helvum* (32, 35, 36) and other species, of which the literature has been thoroughly reviewed in Mbu’u et al. (37). Despite their detection, overall seroprevalence in bats remains low. Interestingly, Pernet et al. (32) detected a 3–4% seroprevalence rate from 497 human blood samples against Hendra virus, GH-74a and NiV, of which almost all positive samples were derived from individuals who reported butchering bats for bushmeat. This finding, combined with the outcomes of our study, underscores the ongoing elevated risk of exposure for individuals engaged in the bushmeat trade.

We also observed seropositivity toward the H17N10 PVs, a bat Influenza A-like virus that contains unconventional HA and NA proteins (38). The evolutionary distinct H17N10 along with H18N11 virus (not screened for in this study) were originally recovered from asymptomatic fruit bats of the Neotropic bat family *Phyllostomidae* (*Sturnira lilium* and *Artibeus planirostris*) in several countries of Central and South America (38–40). These viruses have attracted considerable attention following reports that their entry in host cells is mediated by the conserved trans-species MHC-II proteins, suggesting zoonotic potential (41, 42). So far, bats in Central and S. America, have been found seropositive for H17N10 and H18N11, but not in a study in Central Europe (43), and to our knowledge this is the first report of H17-neutralizing samples in non-Neotropical bat species. Considering the lack of a validated serological assay to screen *E. helvum* bat sera specifically for H17N10, we cannot exclude serological cross-reactivity with heterologous H17-like antigens. Nonetheless, the potential presence of undiscovered H17 or H17-like IAV species in *E. helvum*, such as the ones described in *Phylostomidae* is tantalizing, cannot be ruled out and warrants further examination by unbiased approaches, i.e., metagenomics in subsequent studies.

Although we only detected a single sample positive for neutralizing antibodies against EBOV, similar studies have also reported very low seroprevalence rates in *E. helvum*; 1 out of 262 samples from Ghana (44), and 19 out of 748 samples from Zambia (45). These low numbers could be due to the fact *E. helvum* bat cells have been shown to be refractory toward EBOV infection (46). On the other hand, a report detected much higher seroprevalence of EBOV antibodies in *E. helvum* from Cameroon, with 107 out of 817 positive samples (47). Again, it is vital to consider the possibility of cross-neutralization, not only with other pathogenic species of ebolaviruses but potentially closely related undiscovered filoviruses, which may not be pathogenic in humans such as the newly discovered Bombali virus in Africa, and shares cross-neutralization against EBOV antibodies (48).

There are several limitations to our study. Relative to the size of bat colonies, 304 samples is small and as a result may not accurately represent potential viruses in circulation within the colony. Secondly, the use of PVs alone, without nucleic acid testing does not definitively inform whether the antibodies we detected were generated by infection of the stated virus or a close relative, resulting in cross neutralizing antibodies, which we have detailed in the discussion. Nonetheless, the preliminary nature of our data in this study would suggest that it is worth carrying out a more comprehensive investigation of *E. helvum* bats in Makurdi to assess the potential risks posed to the human and animal population.

In summary, our serological screening of *E. helvum* sera obtained from bats has revealed the presence of antibodies that can neutralize PVs displaying the glycoproteins of several highly pathogenic viruses. The capture and preparation of these bats for human consumption suggests a potential for direct exposure to bat bodily fluids, thereby elevating the risk of cross-species transmission of the viruses and other pathogens. Given that human settlements are encroaching into areas known to harbor large bat colonies, especially in areas that are known for bat roosting, the risk of zoonotic spillover will continue to increase. Therefore, monitoring of bat viruses especially in the large bat populations in Sub-Saharan Africa is crucial in order to better understand the prevalence and transmission of viruses and to mitigate the risk of potential spillover events.

## Data availability statement

The raw data supporting the conclusions of this article will be made available by the authors, without undue reservation.

## Ethics statement

The animal study was approved by Approval to sample bat populations (VDS/194/S.4/11/T/85) was obtained from the Director/Chief Veterinary Officer of Nigeria, Department of Veterinary and Pest Control Services, Federal Ministry of Agriculture and Pest Control Services, Abuja Nigeria. The study was conducted in accordance with the local legislation and institutional requirements.

## Author contributions

DC: Conceptualization, Data curation, Formal analysis, Investigation, Methodology, Resources, Validation, Visualization, Writing – original draft, Writing – review & editing. MM-N: Data curation, Formal analysis, Investigation, Methodology, Validation, Writing – review & editing. BA: Data curation, Formal analysis, Investigation, Methodology, Writing – review & editing. KD: Data curation, Formal analysis, Investigation, Methodology, Writing – review & editing. JDR: Conceptualization, Data curation, Formal analysis, Investigation, Methodology, Writing – review & editing. VA: Data curation, Investigation, Methodology, Resources, Writing – review & editing. CS: Investigation, Resources, Validation, Writing – review & editing. MD: Conceptualization, Data curation, Formal analysis, Methodology, Writing – review & editing. AH: Formal analysis, Investigation, Validation, Visualization, Writing – review & editing. EW: Conceptualization, Investigation, Methodology, Resources, Supervision, Writing – original draft, Writing – review & editing. SS: Investigation, Methodology, Resources, Supervision, Validation, Writing – original draft, Writing – review & editing. EG: Data curation, Formal analysis, Investigation, Methodology, Software, Validation, Visualization, Writing – review & editing. AB: Investigation, Resources, Supervision, Validation, Writing – review & editing. NT: Conceptualization, Data curation, Formal analysis, Investigation, Methodology, Resources, Supervision, Validation, Visualization, Writing – review & editing. IJ: Resources, Writing - review and editing, Validation.
